# An assembled bacterial community associated with *Artemisia annua* L. causes plant protection against a pathogenic fungus

**DOI:** 10.3389/fmicb.2023.1218474

**Published:** 2023-10-09

**Authors:** Yu Wang, Zhan-nan Yang, Shi-qiong Luo

**Affiliations:** ^1^School of Life Sciences, Guizhou Normal University, Guiyang, Guizhou, China; ^2^Key Laboratory for Information System of Mountainous Areas and Protection of Ecological Environment of Guizhou Province, Guizhou Normal University, Guiyang, Guizhou, China

**Keywords:** *Artemisia annua*, assembled bacterial community, pathogenic fungus, plant–bacterium interaction, *Globisporangium ultimum*

## Abstract

The microorganisms associated with a plant influence its growth and fitness. These microorganisms accumulate on the aerial and root surfaces of plants, as well as within the plants, as endophytes, although how the interaction between microorganisms protects the plant from pathogens is still little understood. In the current study, the impact of assembled the bacterial communities against the pathogenic fungus to promote *Artemisia annua* L. growths was investigated. We established a model of bacterium–fungus–plant system. Eight bacterial strains and a fungal pathogen *Globisporangium ultimum* (Glo) were isolated from wild *A. annua* roots and leaves, respectively. We assembled the six-bacteria community (C6: *Rhizobium pusense*, *Paracoccus* sp., *Flavobacterium* sp., *Brevundimonas* sp., *Stenotrophomonas* sp., and *Bacillus* sp.) with inhibition, and eight-bacteria community (C8) composing of C6 plus another two bacteria (*Brevibacillus nitrificans* and *Cupriavidus* sp.) without inhibition against Glo in individually dual culture assays. Inoculation of seedlings with C8 significantly reduced impact of Glo. The growth and disease suppression of *A. annua* seedlings inoculated with C8 + Glo were significantly better than those of seedlings inoculated with only Glo. C8 had more inhibitory effects on Glo, and also enhanced the contents of four metabolites in seedling roots compared to Glo treatment only. Additionally, the inhibitory effects of root extracts from *A. annua* seedlings showed that Glo was most sensitive, the degree of eight bacteria sensitivity were various with different concentrations. Our findings suggested that the non-inhibitory bacteria played a vital role in the bacterial community composition and that some bacterial taxa were associated with disease suppression. The construction of a defined assembled bacterial community could be used as a biological fungicide, promoting biological disease control of plants.

## Introduction

The microorganisms living in close association with plants influence plant growth and fitness (Castrillo et al., 2017; Durán et al., 2018; Herrera et al., 2018; Finkel et al., 2020). In response to the plant and environmentally derived signals, these microorganisms accumulate on or within the plants (Fitzpatrick et al., 2018; Finkel et al., 2019; Thiergart et al., 2020). Antagonistic interactions between beneficial and pathogenic microorganisms are known to play a critical role in shaping the composition of the plant microbiota and in protecting plants from pathogens (Müller et al., 2016; Durán et al., 2018; Chen et al., 2020; Finkel et al., 2020). However, how the mechanisms involved are not fully understood.

A controllable microorganism-plant system can be established on aseptically grown plants which are then inoculated with a defined microbial community (McNulty et al., 2011; Bodenhausen et al., 2014); some research on such model systems has been reported (Berendsen et al., 2012; Mueller and Sachs, 2015; Wei et al., 2015). For example, leaves of the model plant *Arabidopsis thaliana* were inoculated with a controllable and defined bacterial community to characterize how some plant genes shaped the microbiota of the phyllosphere (Finkel et al., 2020). It has been reported that such simplified microbial communities colonizing the plants can protect the plant against invading pathogens. For example, a simplified and controllable synthetic community, made up of eight species of bacteria, could control maize seedling blight caused by *Fusarium verticillioides* by inhibiting fungal colonization and growth (Niu et al., 2017). Some biocontrol bacteria can protect plants from fungal disease: fluorescent pseudomonads can promote plant growth and produce antifungal chemicals to inhibit pathogenic fungi, whereas the rhizobacterium *Variovorax boronicumulans* produces the auxin plant growth regulator indole acetic acid to promote plant growth (Dieter and Geneviève, 2005). However, it is unclear whether different invading pathogenic fungi are controlled by different microbiota of plants.

Beneficial bacterial communities associated with plants can control invading pathogen fungi either directly or indirectly (Berendsen et al., 2012; Bai et al., 2015; Lebeis et al., 2015; Wagner et al., 2016; Thiergart et al., 2019; Shalev et al., 2022). Reports about how altering the composition of the bacterial community can serve as a barrier to protect the host plant from invading pathogen fungi, in which the invading pathogen fungi may not necessarily be killed, have been presented (Xue et al., 2015; Vale et al., 2016; Ventorino et al., 2016). For example, colonization by a simplified four-species bacterial community, composed of three high-abundance and one low-abundance bacterial species, not only protected *Astragalus mongholicus* from root rot disease, but also achieved plant-induced systemic resistance (Li et al., 2021). However, for most plants, our understanding of the mechanisms through which the bacterial microbiota protect host plants from pathogen fungi is limited because of the high complexity of bacteria microbiota–pathogen–plant systems, which include the interactions between the bacterial community and the pathogen, between the pathogen and the plant, and between bacterial community members and the plant.

*Artemisia annua* L., named Qinghao in Chinese, is an annual herbaceous member of the Asteraceae family and is also one of the most important traditional Chinese herbs (Committee of Chinese Pharmacopoeia, 2020), being the main source material of anti-malarial medicines based on artemisinin. *A. annua* is widely distributed all over the world and mainly grown in the southwest of China. In addition, *A. annua* plants can adapt to various conditions (Huang et al., 2010; Liu et al., 2013). *A. annua* is a well-known medicinal herb that has low toxicity and a very effective anti-malarial activity due to the presence of the sesquiterpene lactone artemisinin, the first-line anti-malarial drug recommended by the World Health Organization. There are many medicinal activities of *A. annua* (e.g., anticancer) associated with plant metabolites (including phenolics) (Ferreira et al., 2010; Huang et al., 2010; Lévesque and Peter, 2012). Recent studies of *A. annua* mainly focus on the artemisinin biosynthesis mechanism, with the goal of increasing the yield of artemisinin. A linkage has been reported between rhizosphere microbes and medicinal metabolites in *A. annua* (Luo et al., 2019a). Bacterial and fungal communities were markedly influenced by total organic carbon, available phosphorus, total nitrogen, available potassium, and water-soluble nitrogen (Luo et al., 2019b; Shi et al., 2022). Few studies of microbial influence on *A. annua* growth (e.g., phenotype, growth rate, and physiology) have been reported. Plant growth-promoting bacteria, (e.g., *Sphingobium* and *Sphingomonas*), and the fungal amplicon sequencing variants (ASVs) (e.g., of saprotrophs) were significantly enriched in the *A. annua* rhizosphere (Shi et al., 2022). Furthermore, *A. annua* could assemble the specific root-associated microbial communities with an increased abundance of plant growth-promoting microorganisms and build inter-kingdom co-occurrence networks in the rhizosphere (Shi et al., 2022). It has been reported that endophytic bacteria (43 strains), fungi (12 strains), and actinomycetes (43 strains) were isolated from *A. annua* roots (Tian et al., 2008). However, how the interaction between microbiota in the few studies on microbial influence on *A. annua* growth (e.g., phenotype, physiology, and secondary metabolism) have been reported. How the natural *A. annua* microbial assemblage can inhibit invading pathogens and promote the healthy growth of *A. annua* plants is still poorly understood.

In this study, we examined the inhibitory effect of a defined assembled bacterial community on *A. annua* against the fungal pathogen *Globisporangium ultimum* (OR416213), the cause of *A. annua* root rot. We studied the assembly of the assembled bacterial microbiota community of eight bacterial species, which was isolated from *A. annua* roots, and the response of colonized and non-colonized *A. annua* seedlings to *G. ultimum* infection was investigated. The questions which need to be resolved are: (1) Does the assembled bacterial microbiota have a direct inhibitory effect on the pathogen *G. ultimum*? (2) How does the bacterial microbiota affect *G. ultimum* in the disease-suppressive plant–bacterium interaction? (3) How does the *A. annua* root extract affect the growth of the fungal and bacterial isolates?

To answer these questions, we first isolated a major plant pathogen, *G. ultimum* from *A. annua*, and a number of bacterial strains associated with *A. annua*, and we analyzed the effects of the bacterial isolates against the plant pathogen based on screening and dual culture assays. Afterward, we constructed a six-species assembled bacterial microbiota (C6) in which each strain had an inhibitory effect on the pathogen *G. ultimum*. In addition, we also constructed another, eight-species assembled bacterial microbiota (C8) consisting of the six species described in C6 above plus two bacterial species having no inhibitory effects on the fungal pathogen, *G. ultimum*. We then compared the responses of *A. annua* plants to *G. ultimum* following inoculation with C8, C6 or no bacteria *in vivo* in a greenhouse pot experiment. Ultimately, the C8 assembled bacterial community was selected to examine the plant–bacteria–fungus interaction in promoting healthy growth of *A. annua* in the presence of *G. ultimum*.

This study provided evidence that colonization by the assembled bacterial microbiota can inhibit infection by the pathogen *G. ultimum*, to promote healthy growth of *A. annua*; more importantly, it provided an insight into the potential use of assembled bacterial microbiota as biological fungicide, promoting biological disease control of plants.

## Materials and methods

### Collection of *A. annua* plants

Healthy *A. annua* plants were collected from the wild (26°35′31.0″N, 106°43′09.6″E) by uprooting them randomly on 10 September 2021 in Guiyang, Guizhou Province, Southwest China. The samples were placed separately into sterile plastic bags, and transported to the laboratory at Guizhou Normal University, where they were used for isolation of pathogenic fungi, associated bacteria, and for plantlet regeneration.

### Purification and infection of pathogenic fungi

Pathogenic fungi were isolated from mature leaves of *A. annua* plants according to a slight modification of the method of Melo et al. (2014). The leaf samples were rinsed in running water for 30 min to remove soil particles, etc., then sterilized by immersion in 75% (v/v) alcohol for 30 s, followed by transfer to 0.1% (w/v) HgCl_2_ for 6 min under shake conditions to ensure sufficient contact with the sterilant, then rinsed five times in sterile distilled water for 10 s each time. After the water on the leaf surface was dried using sterile filter paper, the sterilized leaves were cut into 1.5–2.0 cm pieces, and placed on potato dextrose agar (PDA), three leaf pieces per Petri dish. The PDA dishes were incubated at 28°C for 7 days in darkness. When the hyphal tips of the fungus protruded from the inner leaf segments on PDA, they were isolated and purified, then transferred to slants and stored at 4°C for use in subsequent experiments.

To screen for the most effective virulent fungus, infection experiments were performed by adding 1 ml fungal spore suspension liquid (10^6^ CFU/ml) of each of the fungal isolates obtained from the wild *A. annua* plants into sterile soil containing sterile *A. annua* plantlets; after culture for 14 days, the pathogenicity rate of each fungus and the corresponding death rate of plantlets were recorded. The most consistently virulent strain was selected for use as the experimental pathogenic fungus.

### Isolation of endophytic bacteria

Endophytic bacteria were isolated from the roots of *A. annua* seedlings, which were regenerated from the wild-harvested *A. annua* plants collected in Guiyang and grown in the humus soil collected from Changbai Mountains, Jilin Province, China (41°35′N, 127°40′E). After separating from the seedling aerial parts, the roots were shaken vigorously to remove loose soil, then rinsed with sterile water until the adhering soil was completely removed, and sterilized as described earlier for leaves. The sterilized roots were cut into pieces, and then extracted in 0.5 mmol/L phosphate-buffered saline (PBS) by ultrasonic vibration for 10 min. The root extract in PBS was diluted into 10^–5^, 10^–6^, and 10^–7^ concentrations. A 0.1 ml aliquot of each concentration was added and spread onto nutrient agar (NA, containing 5.0 g peptone, 18 g agar, 5.0 g NaCl, 1,000 ml distilled water, pH 7.0–7.2), with five replicate plates for each concentration. All NA plates were incubated at 37°C for 3 days; endogenous bacteria were purified and stored at −18°C prior to the assembled community construction stage.

### Dual culture assays and assembly of bacterial communities

Following the method of Gkarmiri et al. (2015), with minor modifications, dual culture assays of the bacterium–fungus interactions were established in 9-cm diameter Petri dishes containing PDA medium. A 7-mm diameter plug cut from the edge of an actively growing colony of each test fungus was inoculated in the middle of each Petri dish. Fresh cells of endophytic bacteria were streaked in 3-cm long parallel lines on either side of the fungal plug; the concentration of each bacterial suspension was 10^6^ CFU/ml. Control plates with the fungus only were also conducted. After all treatments were incubated at 28°C in darkness for 7 days, fungal hyphae challenged with each endophytic bacterial isolate and from control plates were photographed, and the sizes of the inhibition zones were measured.

Based on the fungal inhibitory effects, a six-species assembled bacterial community (C6) was constructed, to consider the synergistic effect among the bacteria. Two bacterial strains without significant inhibitory effect against the pathogenic fungus was added into C6 to construct an eight-species assembled bacterial community (C8), which was composed of the following eight bacteria strains isolated from *A. annua* roots: *Brevundimonas* sp. Yang 2023, G16 *Flavobacterium* sp., 0410ARD7G4 *Paracoccus* sp., CA34 *Rhizobium pusense*, YFCC6454 *Stenotrophomonas* sp., DE024 *Bacillus* sp., S186B *Brevibacillus nitrificans*, and NBRC102508 *Cupriavidus* sp. The same volume and concentration (10^6^ CFU/ml) of each isolate was mixed together (“C6” and “C8” treatments), and “Glo” represented the pathogenic fungus; other treatments included “C8 + Glo” and “C6 + Glo.” We compared the inhibitory effects of the two bacterial communities (C6 and C8) against the pathogenic fungus by soil pot experiments, and selected the bacterial assembled community with the greater antifungal inhibitory effect for the subsequent disease-control pot experiments.

### DNA extraction, amplification, and sequencing

After the fungal and bacterial isolates were purified and screened for pathogenicity and antifungal effects, respectively, we submitted each to Sangon Biotechnology Co., Ltd. (Shanghai, China) for sequencing. Isolates were transported in an expanded polystyrene foam box containing dry ice to the laboratory of Sangon Biotechnology. The DNA was extracted from bacteria using Ezup Column Bacteria Genomic DNA Purification Kit (SK8255, Sangon Biotechnology, Shanghai, China), and the DNA was extracted from fungus with Ezup Column Fungi Genomic DNA Purification Kit (SK8259, Sangon Biotechnology, Shanghai, China), according to the manufacturer’s protocol. The barcoded primers 7F (5′-CAGAGTTTGATCCTGGCT-3′) and 1540R (5′-AGGAGGTGATCCAGCCGCA-3′) were used to amplify the V3–V4 region 16S rDNA of bacteria; the length of the PCR product was 1,500 bp; the barcoded primers ITS1 (5′-TCCGTAGTGACCTGCGG-3′) and ITS4 (5′-TCCTCCGCTTATTGATATGC-3′) were used to amplify the internal transcribed spacer (ITS) V1–V2 region of fungal rDNA; the length of the PCR product was 600 bp. The PCR amplification procedure was as follows: pre-denaturation at 95°C for 4 min, followed by 30 cycles of denaturation at 94°C for 30 s, annealing at 57°C for 30 s, extension at 72°C for 90 s, repair and extension at 72°C for 10 min, and termination at 4°C. The purified PCR products were sequenced on the Illumina MiSeq platform. The sequence alignment of bacterial 16S rDNA was conducted in the GenBank database,^1^ and the sequence alignment of fungal ITS was conducted by BLAST of the NCBI website (see text footnote 1), and submit the sequence data to the Genbank of NCBI,^2^ at last the accession numbers were got. Phylogenetic trees were constructed by the adjacency method using MEGA11 software, and the bootstrap replication number was set to 1,000.

### Soil preparation and physicochemical properties

Control of the pathogenic fungus *G. ultimum* by the bacterial assembled community was tested *in vivo* using humus soil, which was collected from Changbai Mountains. The organic matter, available nitrogen, available phosphorus, available potassium concentrations, and pH of the soil were 81.75 g/kg, 115.47 mg/kg, 69.92 mg/kg, 86.51 mg/kg, and 6.83, respectively. The soil sampling field had no history of *A. annua* growing there before the soil was collected.

A tissue-culture glass container (height 120 mm and diameter 80 mm) was filled with 100 g soil, and sterilized in an autoclave at 105°C for 1 h. After autoclaving, the soil medium was allowed to cool, a 0.1 g sub-sample of the soil was diluted with sterilized deionized water into different concentration (10^–2^, 10^–3^, 10^–4^, 10^–5^, 10^–6^, and 10^–7^), and then 10^–5^, 10^–6^, and 10^–7^ soil solutions were spread on NA (for culturing bacteria) and PDA (for fungi) plates, and incubated at 28°C for 7 days; if no microbial growth occurred, it meant that the soil was sterilized completely and could be used for pot experiment of disease control. The soil that was not sterilized (natural soil) was used for the disease-control treatment. Before subsequent experiments, all soil medium was supplemented with the same volume of sterile deionized water.

### Preparation of fungal and bacterial suspension liquid

After a fungal colony had grown robustly on a PDA plate, the fungal hyphae was transferred into a 150-ml flask containing 50 ml sterile deionized water, and then incubated at 28°C with constant agitation at 200 rpm for 6 h, a method which was conducive to the spores falling from the mycelium and being suspended in the water, forming the fungal spore suspension, which was diluted with sterile deionized water to achieve a concentration of 10^6^ CFU/ml; this was stored at 4°C for use in the disease-control experiment.

After activation was performed at 25°C in darkness, bacterial colonies on NA plates were each transferred individually with an inoculation loop into a 150-ml flask with 50 ml NA broth, and then incubated at 37°C with constant agitation at 200 rpm for 6 h; eventually, the bacterial NA broth was diluted using sterile deionized water to achieve the concentration of 10^6^ CFU/ml and stored at 4°C for use in the disease-control experiment.

### Soil pot experiment of the effect of a bacterial assembled community against fungal disease

Regenerated tissue of *A. annua*, which was cultured according to the method of Wang et al. (2022), was transplanted into the sterilized soil in containers to get many sterile seedlings, then, the sterile seedlings were transplanted to containers with the sterile humus soil, one seedling per container, and the plants were allowed to grow for 3 days. Subsequently, each container with one sterile seedling was inoculated with 10 ml Glo, C8, or Glo + C8 solutions (10^6^ CFU/ml), with control seedlings being treated with 10 ml of sterile deionized water; every treatment was replicated 15 times, with all the plants being grown at 24°C (12-h light/12-h darkness photoperiod). Fungal disease incidence and plant mortality were recorded every 7 days. The antagonistic capacity of C8 toward the pathogenic fungus was assessed in terms of plant growth, concentrations of chlorophyll a and b, malondialdehyde (MDA), and proline, and activities of the antioxidant enzymes superoxide dismutase (SOD) and peroxidase (POD). Moreover, the concentrations of metabolites (artemisinin, scopoletin, chrysosplenol-D, and chrysosplenetin) of *A. annua* roots were determined after the seedlings were cultured for 7 days after inoculation with Glo.

### Inhibitory effect of *A. annua* extract on microbial isolates

To examine whether *A. annua* affects the growth of the fungal or bacterial isolates used in this study, the inhibitory experiment was conducted. A sample (400 g fresh weight) of *A. annua* roots was cut into pieces which were put into a 1,000-ml flask, to which were added 400 ml methanol; the flask was then sealed and placed on a shaker at 25°C at 200 rpm for 12 h. After ultrasonic extraction for 30 min, the extract was filtered into a round-bottomed flask, and evaporated using a rotary vacuum evaporator and stored in a brown bottle at 4°C.

*Artemisia annua* extract, which was adjusted to a density of 1 mg/ml, was sterile filtered by passage through a bacterial filter membrane into PDA at 50∼60°C into plates and into NA broth into rigid test-tubes (diameter 20 mm, height 200 mm), respectively, to final concentrations of *A. annua* extract in the medium of 0 (control, CK), 3, 6, 12, and 24 mg/ml. After activation was performed, a 7-mm diameter plug cut from the edge of an actively growing colony of the pathogenic fungus *G. ultimum* was inoculated onto a PDA plate containing *A. annua* extract, incubated at 28°C, and the diameter of the fungus colony was recorded after culture for 24 and 48 h. To determine the impact on bacterial growth, 1 ml bacterial suspension (10^6^ CFU/ml) was inoculated into NA broth containing *A. annua* extract, incubated at 37°C with constant agitation at 200 rpm for 24 h, and then the optical density (OD) at 600 nm was determined using spectrophotometry, repeated three times for each concentration.

### Statistical analysis

Excel 2016 was used to calculate the mean values of each treatment in dual culture assays, soil pot incubation experiments and inhibitory experiments of the *A. annua* extract. SPSS 23.0 software (SPSS Inc., Chicago, IL, USA) was used to carry out statistical analysis on experimental data, by two-way ANOVA, with Duncan’s multiple range test being used to determine significant differences among treatments at *P* ≤ 0.5.

## Results

### Isolation and pathogenicity of fungi isolated from *A. annua*

**FIGURE 1:**
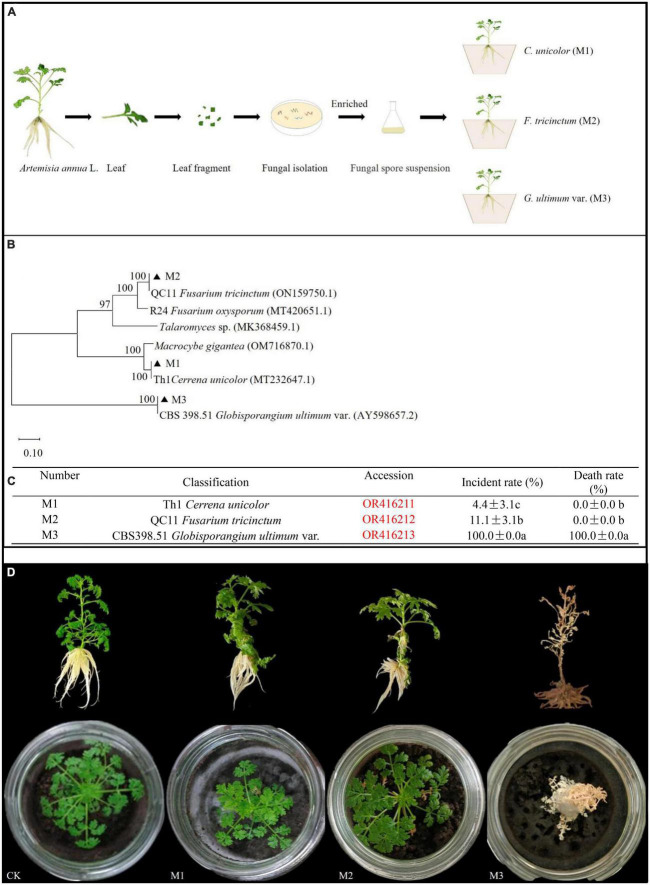
Phylum of fungal isolates from *A. annua* leaves and pathogenic capacity of endogenously fungi pathogens. **(A)** The separation process of pathogenic fungi. **(B)** Phylum of endogenous fungi isolates. **(C)** Plant disease incident rate and death rate in *A. annua* with fungi pathogenic infection, different lower-case letters indicate significant differences among fungi pathogen (*p* < 0.05), same lower-case letters mean no differences. **(D)** The symptoms of plants.

Five different endogenous fungal strains were isolated from *A. annua* leaves (Figure 1A). Through sequence alignment of fungal ITS sequences, they were clustered into three different genera and the accession numbers inquired in Genbank at NCBI website: Th1 *Cerrena unicolor* (OR416211) (M1), QC11 *Fusarium tricinctum* (OR416212) (M2), and CBS398.51 *G. ultimum* (OR416213) (M3) (Figure 1B). According to the inoculation experiment results, *G. ultimum* caused the highest disease incidence (100%) and plant death rate (100%) (Figure 1C). After plants inoculated with *G. ultimum* were cultured for 14 days, *A. annua* plants displayed typical symptoms: all seedings became chlorotic and died; in addition, the surfaces of the soil in the glass container ware covered with flocculent white hyphae (Figure 1D). Therefore, *G. ultimum* was used as the pathogen in the following experiments.

### Screening of antifungal bacteria

**FIGURE 2:**
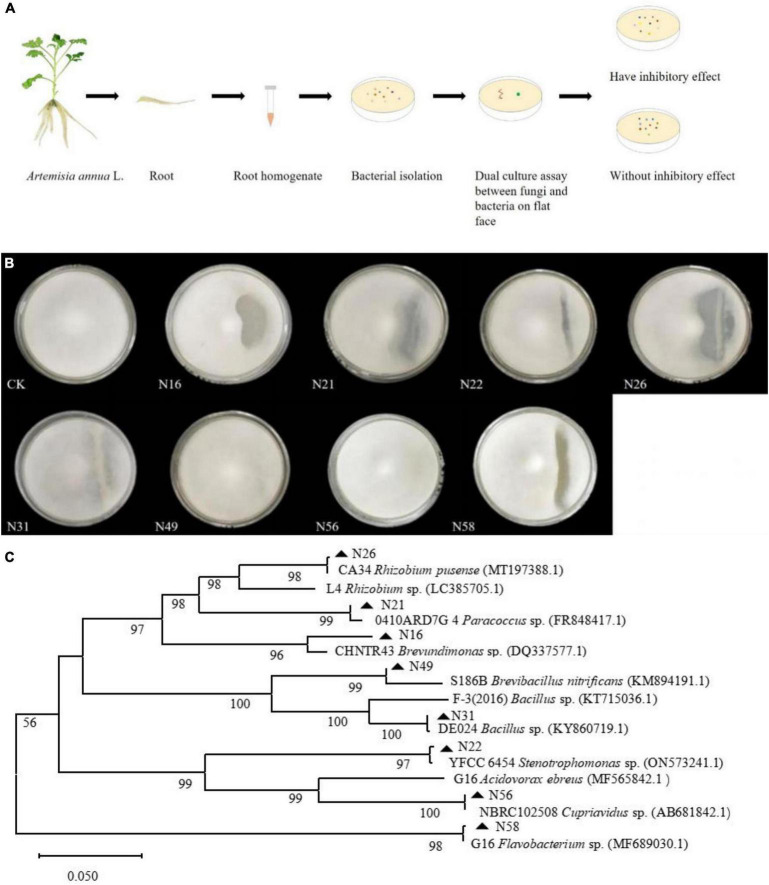
Dual culture assay of every bacterial-fungal interaction and phylum of endogenous bacteria isolates. **(A)** Isolation of endogenous bacteria and dual culture assay. **(B)** Inhibitory effects of eight candidates composing bacteria against on *G. ultimum* var. **(C)** Phylum of endogenous bacteria isolates. CK, *Brevundimonas* sp. Yang 2023, G16 *Flavobacterium* sp., 0410ARD7G4 *Paracoccus* sp., CA34 *Rhizobium pusense*, YFCC6454 *Stenotrophomonas* sp., DE024 *Bacillus* sp., S186B *Brevibacillus nitrificans*, and NBRC102508 *Cupriavidus* sp., respectively.

To screen as many bacterial isolates as possible, more sterile roots were cut into pieces in PBS buffer solution, and spread on NA plates (Figure 2A), and at last 60 endogenous bacterial strains (Supplementary Table 1) were isolated from *A. annua* roots, named N1–N60, After the DNA of each strain was sequenced and the 16S rDNA sequences were aligned, the bacterial strains were clustered into eighteen different genera (Supplementary Figure 1) and the accession numbers inquired in Genbank at NCBI website. Because the similarity of N16 and CHNTR43 *Brevundimonas* sp. is 96.28%, N16 is named as *Brevundimonas* sp. Yang 2023 (OR415840). Dual culture assay of every bacterium–fungus interaction was conducted on PDA plate. Six bacterial taxa, which could each inhibit the growth of the fungus *G. ultimum* (Figure 2B), were selected to be candidates for inclusion in construction of the community of antifungal bacteria (“assembled bacterial community”). The order of inhibitory effect toward the fungus was as follows: CA34 *R. pusense* (OR415843) > 0410ARD7G 4 *Paracoccus* sp. (OR415841) > G16 *Flavobacterium* sp. (OR415847) > *Brevundimonas* sp. Yang 2023 (OR415840) > YFCC6454 *Stenotrophomonas* sp. (OR415842) > DE024 *Bacillus* sp. (OR415844) (Table 1). Although there was no obvious inhibitory zone around S186B *B. nitrificans* (OR415845) and NBRC102508 *Cupriavidus* sp. (OR415846), the white hyphae of the fungus reduced obviously (Figure 2B), and it was believed to have an inhibitory effect on the fungus.

**TABLE 1:** Inhibition of differently disease-resistant bacteria on *Globisporangium ultimum* var. (mean ± standard deviation, *n* = 3).

Number	Name	Accession	Width of inhibition zone
N16	*Brevundimonas* sp. Yang 2023	OR415840	0.82 ± 0.06 b
N58	G16 *Flavobacterium* sp.	OR415847	0.87 ± 0.17 b
N21	0410ARD7G 4 *Paracoccus* sp.	OR415841	1.32 ± 0.10 a
N26	CA34 *Rhizobium pusense*	OR415843	1.57 ± 0.21 a
N22	YFCC6454 *Stenotrophomonas* sp.	OR415842	0.70 ± 0.08 b
N31	DE024 *Bacillus* sp.	OR415844	0.10 ± 0.01 c
N49	S186B *Brevibacillus nitrificans*	OR415845	0.00 ± 0.0 c
N56	NBRC102508 *Cupriavidus* sp.	OR415846	0.00 ± 0.00 c

Different letters in a column indicate significant differences among bacterial strains (*p* < 0.05), same lower-case means no difference, *n* means the repetition.

### Assembly of the community of antifungal bacteria

Because the hyphae of the fungus *G. ultimum* grew very quickly, they reached the 3-cm long parallel lines of antifungal bacteria after only 24 h culture, at which point, some of the bacterial colonies did not grow out of the medium surface, which would affect the measurement of the inhibition zone; after 48 h culture, the hyphae of the fungus *G. ultimum* covered the plate, so that the inhibition zones caused by antifungal bacteria were obvious, and could be measured. The six bacterial isolates with inhibitory effects were mixed in equal volumes of bacterial suspensions (10^6^ CFU/ml) to generate an assembled bacterial community (C6). To investigate whether two bacteria without antifungal activity could contribute to preventing fungal pathogens from infecting *A. annua* plants, we also selected two bacterial isolates, which did not cause visible inhibitory zones in the dual assay, and six bacterial isolates with antifungal activity to construct another assembled bacterial community (C8) (Figure 2B and Supplementary Table 3). We then mixed all the eight species in equal volumes of bacterial suspensions (10^6^ CFU/ml) and constructed assembled bacterial community (C8). A typical phylum picture of the eight bacterial isolates is displayed in Figure 2C.

### Screening of assembled bacterial communities against a pathogenic fungus

After *A. annua* plants were treated with *G. ultimum* and either or none of the two assembled communities (C6, C8, or CK) in an *in vivo* pot experiment for 14 days, those plants inoculated with C6 or neither community (CK) all died, although the six bacterial species in C6 each had an inhibitory effect on the pathogenic fungus *in vitro*. In contrast, the plants inoculated with the eight bacterial species of C8 grew vigorously in the presence of *G. ultimum*. So, the C8 community was screened for the subsequent pot experiment (Supplementary Figure 2).

### Effects of the assembled bacterial community against fungal disease

**FIGURE 3:**
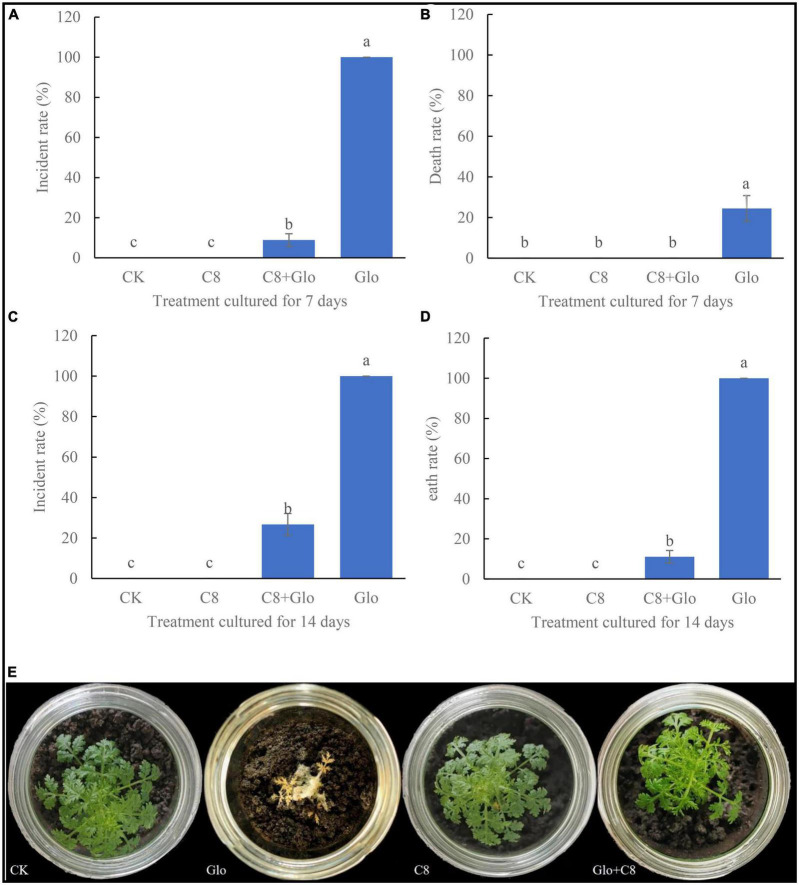
**(A)** Incidental rate of seedling treated with sterile water CK, *G. ultimum* var. Glo, assembled community C8 and C8 + Glo after cultured for 7 days. **(B)** Death rate cultured for 7 days. **(C)** Incidental rate cultured for 14 days. **(D)** Death rate cultured for 14 days. Different letters in a column indicate significant differences among bacterial strains (*p* < 0.05), same lower-case means no difference. **(E)** The pictures of seedling cultured for 14 days in the treatments.

After sterile seedlings were inoculated with *G. ultimum* for 7 or 14 days, the disease incidence rate were 100 and 100% (Figures 3A, C), and the average death rates were 24.5 and 100%, respectively (Figures 3B, D). In addition, after culture for 7 days, the soil surfaces were covered with a large amount of white hypha, with some hyphae climbing up the aerial parts of the seedlings (Supplementary Figure 2), until the seedling was all entwined by the hyphae and could not stand; at this point, the leaves of the seedling dehydrated and became yellow, and seedling growth was obviously inhibited. After culture for 14 days, the roots rotted and the aerial parts became completely dry (Figure 3E). However, after sterile seedlings were inoculated with the assembled community C8 and *G. ultimum* simultaneously for 7 and 14 days, the average disease incidence rates decreased by 91 and 73.3%, respectively (Figures 3A, C), and the average death rates declined by 24.5 and 89.9%, respectively (Figures 3B, D). At the same time, the plants inoculated with the eight-species assembled bacterial community (C8) grew similarly to the control (CK) seedlings (Figure 3E).

### The growth of seedlings colonized by an assembled bacterial community in response to fungal infection

**FIGURE 4:**
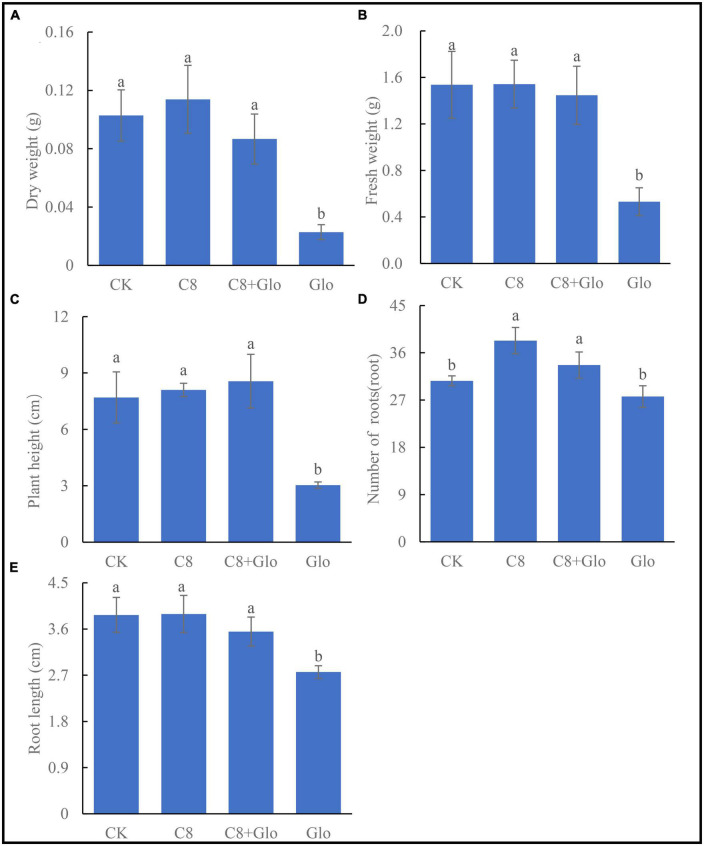
The seedling growth of bacteria assembled community against fungal disease. **(A)** Dry height, **(B)** fresh weight, **(C)** plant height, **(D)** number of roots, and **(E)** root length of *A. annua* plants cultured for 14 days. Different letters in a bar diagram indicate significant differences among strains (*p* < 0.05), same lower-case means no difference.

After *A. annua* plants were inoculated with *G. ultimum* for 14 days, the mean dry weight, fresh weight, plant height, and root length significantly decreased (relative to CK) by 77.8, 65.4, 60.6, and 28.8%, respectively (*p* < 0.05) (Figures 4A-C, E). However, when the plants were inoculated with C8 + Glo, the average number of the plant roots increased significantly (relative to CK), whereas the other indexes of plant growth were not significantly different from those in the CK treatment. When the plants were inoculated with C8 only, they grew better than CK seedlings, although the only significant difference was with respect to the mean number of roots (Figure 4D). These results showed that the assembled bacterial community C8 could also improve the growth of non-diseased *A. annua* plants.

### Physiological indexes of seedlings inoculated with an assembled bacterial community and *G. ultimum*

**FIGURE 5:**
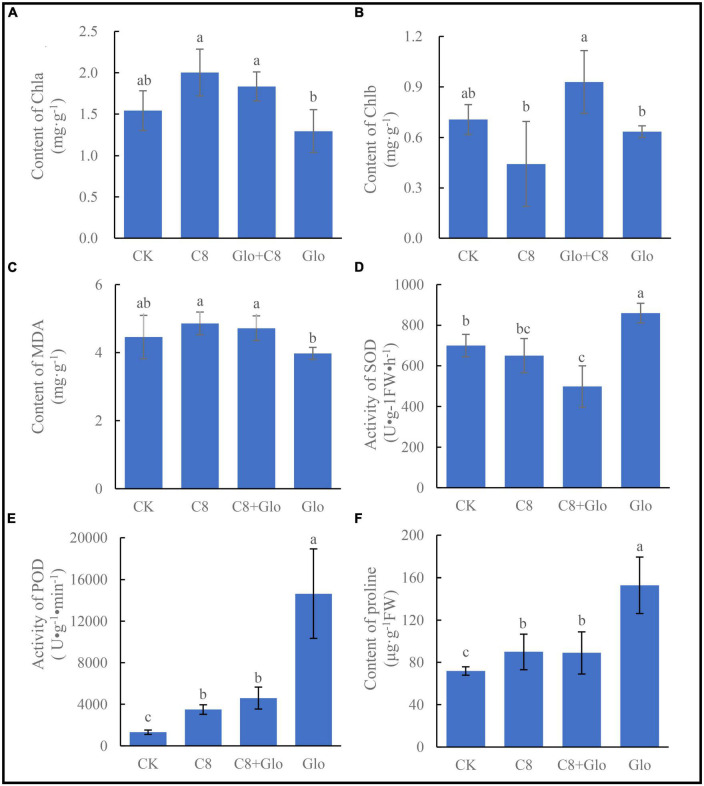
The seedling physiological indexes of bacteria assembled community against fungal disease. **(A)** The chlorophyll *a* (Chla) content, **(B)** the chlorophyll *b* (Chlb) content, **(C)** content of MDA, **(D)** activity of SOD, **(E)** activity of POD, and **(F)** the proline contents. Different letters in a bar diagram indicate significant differences among bacterial strains (*p* < 0.05), same lower-case means no difference.

Compared with control (CK) uninoculated seedlings, the chlorophyll *a* (Chla) concentration of *A. annua* plants inoculated with C8 and C8 + Glo increased, whereas the concentration in seedlings inoculated with *G. ultimum* (Glo) decreased although there no significant difference between the CK and inoculation treatments, and the Chla concentrations of seedlings inoculated with C8 and C8 + Glo were significantly higher than those inoculated with only Glo (Figure 5A). The chlorophyll *b* (Chlb) concentration also varied with different treatments, but there were no significant differences among the CK, C8, and Glo treatments (Figure 5B). Surprisingly, the concentration of MDA, which reflects damage due to oxidative stress, was lower in Glo than in C8 + Glo (Figure 5C). However, compared with CK, the activities of the antioxidant enzymes SOD and especially POD treated with Glo increased significantly 160.04 U⋅g^–1^FW⋅h^–1^ and 13,330.30 U⋅g^–1^⋅min^–1^, respectively; the SOD activities treated with C8 and C8 + Glo decreased 49.51 and 201.51 U⋅g^–1^FW⋅h^–1^, respectively, whereas POD activities treated with C8 and C8 + Glo increased significantly 2,181.64 and 3,285.27 U⋅g^–1^⋅min^–1^ (Figures 5D, E). The proline concentrations varied similarly to POD activities, significantly increasing 81.02, 18.11, and 17.15 μg⋅g^–1^ FW (Figure 5F).

### Metabolite concentrations in seedlings inoculated with the assembled bacterial community and *G. ultimum*

**FIGURE 6:**
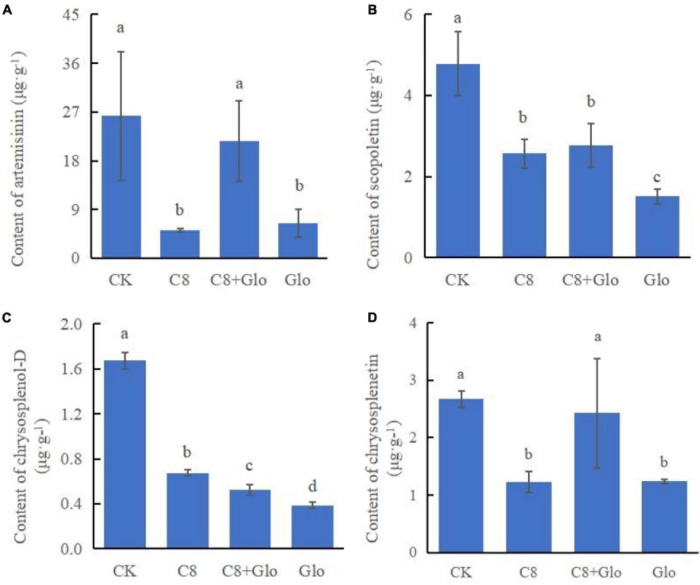
Metabolite contents of the seedling in bacteria assembled community against fungal disease. The contents of **(A)** artemisinin, **(B)** scopoletin, **(C)** chrysosplenol-D, and **(D)** chrysosplenetin of *A. annua* roots after the seedlings were cultured for 7 days. Different letters in a bar diagram indicate significant differences among strains (*p* < 0.05), same lower-case means no difference.

A study was carried out on the concentrations of the main metabolites to determine whether colonization by the assembled bacterial community affected the main metabolites; the metabolite concentrations of *A. annua* roots were measured after the plants were cultured for 7 days. The results showed that the concentrations of the four tested metabolites of seedlings treated with C8, C8 + Glo, and Glo were significantly lower than those in CK seedlings, except for chrysosplenetin and artemisinin treated by C8 + Glo. Compared with Glo, the concentrations of the four metabolites in seedlings inoculated with C8 + Glo increased significantly, and the metabolite concentrations in seedlings inoculated with C8 increased except for artemisinin (Figures 6A-D).

### *In vitro* effects of *A. annua* root extract on the pathogenic fungus and beneficial bacteria

**FIGURE 7:**
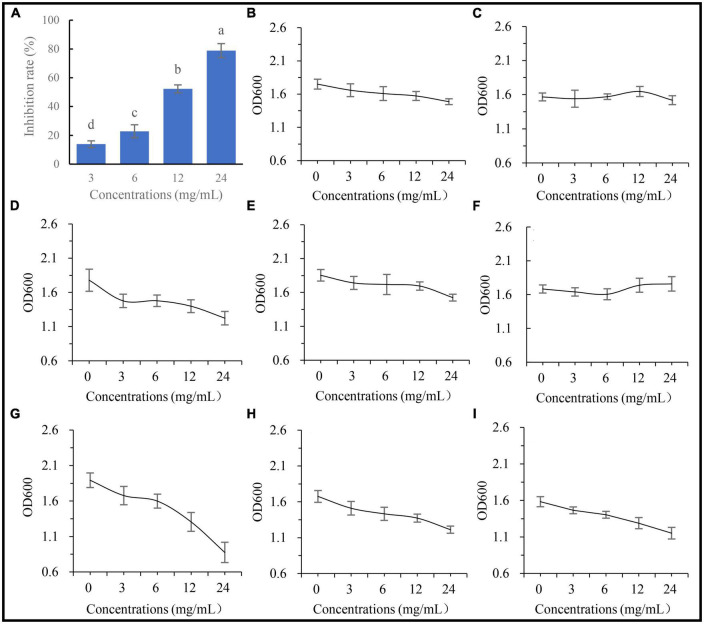
Affection of extracting solution from *A. annua* on pathogenic fungus and bacteria. **(A)** Inhibition rates of extracting solution on *G. ultimum* var. The OD600 of extracting solution against **(B)** *Brevundimonas* sp. Yang 2023, **(C)** G16 *Flavobacterium* sp., **(D)** 0410ARD7G4 *Paracoccus* sp., **(E)** CA34 *Rhizobium pusense*, **(F)** YFCC6454 *Stenotrophomonas* sp., **(G)** DE024 *Bacillus* sp., **(H)** S186B *Brevibacillus nitrificans*, and **(I)** NBRC102508 *Cupriavidus* sp.

To examine whether the metabolites of *A. annua* affect the fungal and bacterial strains, five concentrations of the root extracts were added to the fungal and bacterial culture media, then inoculated with the fungus and the bacterial isolates, respectively, and cultured for 24 h. The results showed that the inhibitory effects of the root extract on *G. ultimum* increased with increasing extract concentrations (Figure 7A), and the diameters of the fungus were negatively correlated with the extract concentrations, and the regression equation was *y* = 7.07 − 0.24x, *R*^2^ = 0.97, *p* < 0.05. In contrast to *G. ultimum*, the bacterial isolates were differentially sensitive to the root extract concentration; isolates 0410ARD7G4 *Paracoccus* sp., DE024 *Bacillus* sp., S186B *B. nitrificans*, NBRC102508 *Cupriavidus* sp., *Brevundimonas* sp. Yang 2023, and CA34 *R. pusense* began to be significantly inhibited at 3.0, 6.0, 6.0, 6.0, 12.0, and 24.0 mg/ml extract concentrations, respectively (Figures 7D, G-I, B, E, respectively), although it is worth noting that YFCC6454 *Stenotrophomonas* sp. and G16 *Flavobacterium* sp. were not affected by all extract concentrations (Figures 7F, C). The order of the degree of sensitivity of the microbial strains to the extracts was *G. ultimum* ∼ DE024 *Bacillus* sp. > 0410ARD7G4 *Paracoccus* sp. ∼ S186B *B. nitrificans* ∼ NBRC102508 *Cupriavidus* sp. > *Brevundimonas* sp. Yang 2023 > CA34 *R. pusense* > G16 *Flavobacterium* sp. ∼ YFCC6454 *Stenotrophomonas* sp. (Figures 7A, G, D, H, I, B, E, C, F, respectively).

## Discussion

### Existence and isolation of the *A. annua* pathogenic fungus

Under natural conditions, all plants suffer from abiotic and biotic stresses, such as drought, waterlogging, nutrient deficiency, nematodes, and pathogenic fungi (Farh et al., 2018; Yuan et al., 2018). *A. annua* is a pioneer plant, the only source of the antimalarial drug artemisinin (Committee of Chinese Pharmacopoeia, 2020), and is tolerant to soil nutrient deficiency (Luo et al., 2019b). The current study is the first report that *A. annua* plants are affected by pathogenic microorganisms (Figure 1). There are microorganisms pathogenic on *A. annua*, such as *G. ultimum*, but possibly natural associations between *A. annua* and bacterial communities achieve suppression of such pathogens.

### Isolation and assembly complex of antifungal bacteria associated with the plants achieve adaptation to the environment

The composition of the microbial community in the rhizosphere of plants, such as maize, paddy rice, wheat, and so on, has been extensively studied (Edwards et al., 2015; Schlemper et al., 2018; Walters et al., 2018). Some members of the bacterial community which directly influenced *A. mongholicus* growth and improved the adaptation of the plants to the environment have been reported (Li et al., 2021). However, it is unclear how *A. annua* plants associate with protective microbes to improve their adaptation to different environments. Because we discovered that the sterile plantlets, which were regenerated from *A. annua* collected in Guiyang, could not grow directly in sterile humus soil from Changbai Mountains when inoculated with *G. ultimum*, it may be that geographic differences cause such results. When we transferred the sterile plantlets to glass containers with non-sterile humus soil inoculated with *G. ultimum* and cultured them for a period of time, the plants were alive and grew vigorously, indicating that the soil microbes were beneficial to the growth of *A. annua* plants; a previous study had reported that plant root systems have a strong selective effect on microorganisms in the surrounding soil (Dibbern et al., 2014), supporting our strategy of isolating bacteria from the roots of *A. annua* plants grown in natural humus soil from Changbai Mountains. After screening of bacterium–fungus interactions with the dual culture assay, bacterial isolates with antifungal activity were selected.

A previous study had reported that a simplified four-species bacterial community, which was composed of three high-abundance bacteria (*Stenotrophomonas* sp., *Rhizobium* sp., and *Ochrobactrum* sp.) and one low-abundance bacterium (*Advenella* sp.), had inhibitory effects on a root rot disease of *A. mongholicus* (Li et al., 2021). A disease-induced assembly of a three-species bacterial community (*Microbacterium* sp., *Stenotrophomonas* sp., and *Xanthomonas* sp.) interacted in biofilm formation to reduce disease incidence (Berendsen et al., 2018). Investigation of bacteria and fungi abundances by amplicon sequencing revealed that the abundance of *Sphingomonas* and *Sphingobium* (both plant growth-promoting bacteria) and saprotrophic fungi in the rhizosphere soil of *A. annua* plants increased dramatically (Shi et al., 2022), but specific isolates were not purified. In our study, 60 bacterial strains were obtained from *A. annua* roots belonging to 18 bacterial genera, from which eight bacterial genera were selected to make up an antifungal assembled bacterial community with activity against Glo. We compared the seedlings inoculated with C8 + Glo and C6 + Glo; the C6 community was composed of six antifungal bacterial isolates individually inhibiting the pathogenic fungus Glo, and the C8 community was composed of C6 plus another two bacterial isolates, S186B *B. nitrificans* and NBRC102508 *Cupriavidus* sp., which had no inhibitory effect on Glo. The results showed that the incidence of disease and the death rate of the seedlings inoculated with C8 + Glo after culture 14 days were significantly lower than in C6 + Glo (Supplementary Figure 2). The results explained that antifungal and non-antifungal bacteria in the assembled community synergistically enhanced plant disease-suppressive activity.

### Effect on plants of assembled bacterial community against *G. ultimum*

An earlier study reported that an assembled bacterial community influenced the root growth of model plant *Arabidopsis*, with one single bacterial genus, *Variovorax*, of the assembled community manipulating plant hormone levels to achieve the positive effects of the assembled root community on root growth (Finkel et al., 2020). In the current study, the eight-species community had a robust effect on *A. annua* plants infected by the pathogen *G. ultimum*. Similarly, the concentrations of four metabolites of *A. annua* roots inoculated with C8 + Glo were significantly higher than in those inoculated with Glo, with the concentrations of artemisinin and scopoletin being significantly higher with C8 + Glo than with C8, suggesting that the eight-species assembled bacterial community enhanced the levels of *A. annua* metabolites under disease-suppressive condition. It has been reported that artemisinin has anti-fungal and anti-bacterial activities in addition to anti-malarial activity (Huang et al., 1993; Appalasamy et al., 2014), whereas scopoletin, chrysosplenol-D and chrysosplenol all had antioxidant activities to reduce the impact of oxidative stress (Lai et al., 2007; Brisibe et al., 2009; Yang et al., 2009; Ferreira et al., 2010), which could suggest that they would contribute to increased disease suppression in *A. annua*.

The increased disease resistance of C8-treated *A. annua* was also demonstrated by physiological indexes, with the concentrations of Chla and Chlb treated with C8 + Glo being markedly higher than when treated with Glo alone (Figures 5A, B), suggesting that the eight-species community had a robust beneficial effect on plant chlorophyll levels under disease attack. However, the POD and SOD activities were higher in Glo than in C8 + Glo (Figures 5D, E), possibly reflecting that the plants were responding to the increased oxidative stress associated with disease; unexpectedly, the concentration of the end-product of the stress, MDA, was lower in diseased plants.

### Mechanism supposition of assembled bacteria community against fungi pathogen

After inoculating sterile seedlings with C8 + Glo in sterile soil and culturing for 7 days, the eight bacteria were traced by 16S RNA sequencing. For more sensitivity and specificity, ASVs were used to quantified the abundances of the endogenetic bacteria in roots (Callahan et al., 2016, 2019). ASV values ranged from 1.00 to 6,952.33 in C8 treatment, NBRC102508 *Cupriavidus* sp. was the highest, followed by CA34 *R. pusense*; and ASV varied from 1.67 to 7,454.33 in C8 + Glo treatment, but CA34 *R. pusense* was the highest, followed by NBRC102508 *Cupriavidus* sp., and G16 *Flavobacterium* sp. was the lowest in both treatments (Supplementary Table 2). It was discovered that eight bacteria could colonized in endosphere of plant roots, although the abundances of the endogenetic bacteria were different. In addition, the crude extract from C8 culture solution and dual cultural assay exhibited that C8 had inhibitory effect on Glo (Supplementary Figures 3, 4), which suggested that secretions from eight bacteria complex prevented pathogen Glo from growth. It could be seen that such functionally assembled community could be used as biological fungicides to help the growth of plants.

### Different effects of *A. annua* root extract against fungi and bacteria

Other plant metabolites, such as coumarins and phenolic acids (Brown, 1992; Rice-Evans et al., 1996; Ferreira et al., 2010) have antioxidant activities, whereas some volatile components have anti-microbial activities (Juteau et al., 2002; Wu et al., 2007). To test whether such components affect the growth of fungal and bacterial isolates, five different concentrations of root extracts were tested for activity against bacterial and fungal growth *in vitro*. After culture for 24 h, the extract was significantly more inhibitory toward Glo than toward any one of the eight bacterial isolates (Figures 7B–I).

In all, the assembled bacterial community from roots of *A. annua* plants protected the plants against the Glo pathogen. In turn, the root extract of *A. annua* plants had inhibitory activity toward the pathogenic fungus, while the concentrations of some potentially anti-fungal and anti-stress metabolites were lower in C8 + Glo than in Glo. These findings suggested that *A. annua* plants were associated with a natural root bacterial community which suppressed the effects of the pathogen and this effect could be replicated by the assembled community of the *A. annua* root eight-bacteria. The specific mechanism of this bacteria–plant interactions deserve to be studied in more detail in the future.

## Conclusion

The assemblage of six bacterial species with antifungal activity and two bacterial species without effectively protected the plants against Glo as an assembled community applied to sterile plants. These findings suggested that microbes with various ecological functions could be assembled into assembled communities for plant disease suppression. Such functional complex communities could be applied for use as biological fungicides, promoting biological disease control of plants.

## Data availability statement

The original contributions presented in this study are included in this article/Supplementary material, further inquiries can be directed to the corresponding authors.

## Author contributions

YW conducted the experiments, calculated the data, and prepared the figures with direction of Z-nY and S-qL. S-qL wrote and edited the manuscript. Z-nY reviewed and edited the final manuscript. All authors contributed to the article and approved the submitted version.

## Footnotes

1
https://submit.ncbi.nlm.nih.gov/

2
https://blast.ncbi.nlm.nih.gov/Blast.cgi?PROGRAM=blastn&PAGE_TYPE=BlastSearch&LINK_LOC=blasthome

## References

[B1] Appalasamy S. Lo K. Y. Ch’ng S. J. Nornadia K. Othman S. A. Chan L. (2014). Antimicrobial activity of artemisinin and precursor derived from in vitro plantlets of *Artemisia annua* L. *Biomed. Res. Int.* 2014:215872. 10.1155/2014/215872 24575401 PMC3915762

[B2] Bai Y. Müller D. B. Srinivas G. Garrido-Oter R. Potthoff E. Rott M. (2015). Functional overlap of the *Arabidopsis* leaf and root microbiota. *Nature* 528 364–363. 10.1038/nature16192 26633631

[B3] Berendsen R. L. Pieterse C. M. Bakker P. A. (2012). The rhizosphere microbiome and plant health. *Trends Plant Sci*. 17 478–486. 10.1016/j.tplants.2012.04.001 22564542

[B4] Berendsen R. L. Vismans G. Yu K. Song Y. de Jonge R. Burgman W. P. (2018). Disease-induced assemblage of a plant-beneficial bacterial consortium. *ISME J.* 12 1496–1507. 10.1038/s41396-018-0093-1 29520025 PMC5956071

[B5] Bodenhausen N. Bortfeld-Miller M. Ackermann M. Vorholt J. A. (2014). A synthetic community approach reveals plant genotypes affecting the phyllosphere microbiota. *PLoS Genet*. 10:e1004283. 10.1371/journal.pgen.1004283 24743269 PMC3990490

[B6] Brisibe E. A. Umoren U. E. Brisibe F. Magalhaees P. M. Ferreira J. F. S. Luthria D. (2009). Nutritional characterisation and antioxidant capacity of different tissues of *Artemisia annua* L. *Food Chem.* 115 1240–1246. 10.1016/j.foodchem.2009.01.033

[B7] Brown G. D. (1992). Two new compounds from *Artemisia annua*. *J. Nat. Prod.* 55 1756–1760. 10.1021/np50090a006

[B8] Callahan B. J. McMurdie P. J. Rosen M. J. Han A. W. Johnson A. J. Holmes S. P. (2016). DADA2: High-resolution sample inference from Illumina amplicon data. *Nat. Methods* 13 581–583. 10.1038/nmeth.3869 27214047 PMC4927377

[B9] Callahan B. J. Wong J. Heiner C. Oh S. Theriot C. M. Gulati A. S. (2019). High-throughput amplicon sequencing of the full-length 16S rRNA gene with single-nucleotide resolution. *Nucleic Acids Res.* 47:e103. 10.1093/nar/gkz569 31269198 PMC6765137

[B10] Castrillo G. Teixeira P. J. P. L. Paredes S. H. Law T. F. de Lorenzo L. Feltcher M. E. (2017). Root microbiota drive direct integration of phosphate stress and immunity. *Nature* 543 513–518. 10.1038/nature21417 28297714 PMC5364063

[B11] Chen T. Nomura K. Wang X. Sohrabi R. Xu J. Yao L. (2020). A plant genetic network for preventing dysbiosis in the phyllosphere. *Nature* 580 653–657. 10.1038/s41586-020-2185 32350464 PMC7197412

[B12] Committee of Chinese Pharmacopoeia (2020). *Chinese pharmacopoeia*, 1st Edn. Beijing: China Medical Science and Technology Press.

[B13] Dibbern D. Schmalwasser A. Lueders T. Totsche K. U. (2014). Selective transport of plant root-associated bacterial populations in agricultural soils upon snowmelt. *Soil Biol. Biochem.* 69 187–196. 10.1016/j.soilbio.2013.10.040

[B14] Dieter H. Geneviève D. (2005). Biological control of soil-borne pathogens by *Fluorescent pseudomonads*. *Nat. Rev. Microbiol*. 3 307–319. 10.1038/nrmicro1129 15759041

[B15] Durán P. Thiergart T. Garrido-Oter R. Agler M. Kemen E. Schulze-Lefert P. (2018). Microbial interkingdom interactions in roots promote Arabidopsis survival. *Cell* 175 973–983.e14. 10.1016/j.cell.2018.10.020 30388454 PMC6218654

[B16] Edwards J. Johnson C. Santos-Medellin C. Lurie E. Podishetty N. K. Bhatnagar S. (2015). Structure, variation, and assembly of the root-associated microbiomes of rice. *Proc. Natl. Acad. Sci. U.S.A.* 112 E911–E920. 10.1073/pnas.1414592112 25605935 PMC4345613

[B17] Farh M. E. Kim Y. Kim Y. J. Yang D. (2018). Cylindrocarpon destructans/Ilyonectria radicicola-species complex: Causative agent of ginseng root-rot disease and rusty symptoms. *J. Ginseng Res*. 42 9–15. 29348716 10.1016/j.jgr.2017.01.004 PMC5766697

[B18] Ferreira J. F. S. Luthria D. L. Sasaki T. Heyerick A. (2010). Flavonoids from *Artemisia annua* L. as antioxidants and their potential synergism with artemisinin against malaria and cancer. *Molecules* 15 3135–3170. 10.3390/molecules15053135 20657468 PMC6263261

[B19] Finkel O. M. Salas-González I. Castrillo G. Conway J. M. Law T. F. Teixeira P. J. P. L. (2020). A single bacterial genus maintains root growth in a complex microbiome. *Nature* 587 103–108. 10.1038/s41586-020-2778-7 32999461 PMC10329457

[B20] Finkel O. M. Salas-González I. Castrillo G. Spaepen S. Law T. F. Teixeira P. J. P. L. (2019). The effects of soil phosphorus content on plant microbiota are driven by the plant phosphate starvation response. *PLoS Biol.* 17:e3000534. 10.1371/journal.pbio.3000534 31721759 PMC6876890

[B21] Fitzpatrick C. R. Copeland J. Wang P. W. Guttman D. S. Kotanen P. M. Johnson M. T. J. (2018). Assembly and ecological function of the root microbiome across angiosperm plant species. *Proc. Natl. Acad. Sci. U.S.A.* 115 E1157–E1165. 10.1073/pnas.1717617115 29358405 PMC5819437

[B22] Gkarmiri K. Finlay R. D. Alström S. Thomas E. Cubeta M. A. Högberg N. (2015). Transcriptomic changes in the plant pathogenic fungus *Rhizoctonia solani* AG-3 in response to the antagonistic bacteria *Serratia proteamaculans* and *Serratia plymuthica*. *BMC Genomics* 16:630. 10.1186/s12864-015-1758-z 26296338 PMC4546130

[B23] Herrera P. S. Gao T. Law T. F. Finkel O. M. Mucyn T. Teixeira P. J. P. L. (2018). Design of synthetic bacterial communities for predictable plant phenotypes. *PLoS Biol*. 16:e2003962. 10.1371/journal.pbio.2003962 29462153 PMC5819758

[B24] Huang L. Liu J. F. Liu L. X. Li D. F. Zhang Y. Nui H. Z. (1993). Study on the antipyretic and anti-inflammatory effects of *Artemisia annua* L. China. *J. Chin. Mater. Med.* 18 44–48. 8323686

[B25] Huang L. Xie C. Duan B. Chen S. (2010). Mapping the potential distribution of high artemisinin-yielding *Artemisia annua* L. (Qinghao) in China with a geographic information system. *Chin. Med.* 5:18. 10.1186/1749-8546-5-18 PMC2886037 20470438

[B26] Juteau F. Masotti V. Bessière J. M. Dherbomez M. Viano J. (2002). Antibacterial and antioxidant activities of *Artemisia annua* essential oil. *Fitoterapia* 73 532–535. 10.1016/s0367-326x(02)00175-2 12385883

[B27] Lai J. P. Lim Y. H. Su J. Shen H. Ong C. N. (2007). Identification and characterization of major flavonoids and caffeoylquinic acids in three Compositae plants by LC/DAD-APCI/MS. *J. Chromatogr. B Analyt. Technol. Biomed. Life Sci.* 848 215–225. 10.1016/j.jchromb.2006.10.028 17084113

[B28] Lebeis S. L. Paredes S. H. Lundberg D. S. Breakfield N. Gehring J. McDonald M. (2015). Salicylic acid modulates colonization of the root microbiome by specific bacterial taxa. *Science* 349 860–864. 10.1126/science.aaa8764 26184915

[B29] Lévesque F. Peter H. S. (2012). Continuous-flflow synthesis of the antimalaria drug artemisinin. *Angew. Chem. Int. Edit*. 51 1706–1709. 10.1002/anie.201107446 22250044

[B30] Li Z. Bai X. Jiao S. Li Y. Li P. Yang Y. (2021). A simplified synthetic community rescues *Astragalus mongholicu*s from root rot disease by activating plant-induced systemic resistance. *Microbiome* 9:217. 10.1186/s40168-021-01169-9 34732249 PMC8567675

[B31] Liu H. Tian X. Zhang Y. Wang C. Jiang H. (2013). The discovery of *Artemisia annua* L. in the Shengjindian cemetery, Xinjiang, China and its implications for early uses of traditional Chinese herbal medicine qinghao. *J. Ethnopharmacol.* 146 278–286. 10.1016/j.jep.2012.12.044 23295167

[B32] Luo S. Zhao C. Yang Z. Di S. Zheng Z. Hu J. (2019b). Correlation analysis of nutrients, enzymes, and microbial biomass in soils phenolics of *Artemisia annua* L. *Pak. J. Agri. Sci.* 56 171–178. 10.21162/PAKJAS/19.5605

[B33] Luo S. Zhao C. Yang Z. Hu J. Di S. (2019a). Soil microbes and medical metabolites of *Artemisia annua* L. along altitudinal gradient in Guizhou Karst terrains of China. *J. Plant Interact*. 14 167–176. 10.1080/17429145.2019.1602886

[B34] McNulty N. P. Yatsunenko T. Hsiao A. Faith J. J. Muegge B. D. Goodman A. L. (2011). The impact of a consortium of fermented milk strains on the gut microbiome of gnotobiotic mice and monozygotic twins. *Sci. Transl. Med.* 3:106ra106. 10.1126/scitranslmed.3002701 22030749 PMC3303609

[B35] Melo I. S. Santos S. N. Rosa L. H. Parma M. M. Silva L. J. Queiroz S. C. N. (2014). Isolation and biological activities of an endophytic Mortierella alpina strain from the Antarctic moss Schistidium antarctici. *Extremophiles* 18 15–23. 24126742 10.1007/s00792-013-0588-7

[B36] Mueller U. G. Sachs J. L. (2015). Engineering microbiomes to improve plant and animal health. *Trends Microbiol*. 23 606–617. 10.1016/j.tim.2015.07.009 26422463

[B37] Müller D. B. Vogel C. Bai Y. Vorholt J. A. (2016). The plant microbiota: Systems-level insights and perspectives. *Annu. Rev. Genet.* 50 211–223. 10.1146/annurev-genet-120215-034952 27648643

[B38] Niu B. Paulson J. N. Zheng X. Kolter R. (2017). Simplified and representative bacterial community of maize roots. *Proc. Natl. Acad. Sci. U.S.A.* 114 E2450–E2459. 10.1073/pnas.1616148114 28275097 PMC5373366

[B39] Rice-Evans C. A. Miller N. J. Paganga G. (1996). Structure-antioxidant activity relationships of flavonoids and phenolic acids. *Free Radic. Biol. Med*. 20 933–956. 10.1016/0891-5849(95)02227-9 8743980

[B40] Schlemper T. R. van Veen J. A. Kuramae E. E. (2018). Co-variation of bacterial and fungal communities in different sorghum cultivars and growth stages is soil dependent. *Microb. Ecol.* 76 205–214. 10.1007/s00248-017-1108-6 29147973 PMC6061463

[B41] Shalev O. Karasov T. L. Lundberg D. S. Ashkenazy H. Ayutthaya P. P. N. Weigel D. (2022). Commensal *Pseudomonas* strains facilitate protective response against pathogens in the host plant. *Nat. Ecol. Evol*. 6 383–396. 10.1038/s41559-022-0167 35210578 PMC8986537

[B42] Shi Y. Pan Y. Li X. Zhu Z. Fu W. Hao G. (2022). Assembly of rhizosphere microbial communities in *Artemisia annua*: Recruitment of plant growth-promoting microorganisms and inter-kingdom interactions between bacteria and fungi. *Plant Soil* 470 127–139. 10.1007/s11104-021-04829-9

[B43] Thiergart T. Durán P. Ellis T. Vannier N. Garrido-Oter R. Kemen E. (2020). Root microbiota assembly and adaptive differentiation among European *Arabidopsis* populations. *Nat. Ecol. Evol*. 4 122–131. 10.1038/s41559-019-1063-3 31900452

[B44] Thiergart T. Zgadzaj R. Bozsóki Z. Garrido-Oter R. Radutoiu S. Schulze-Lefert P. (2019). *Lotus japonicus* symbiosis genes impact microbial interactions between symbionts and multiking domcommensal communities. *mBio* 10 e01833–19. 10.1128/mBio.01833-19 31594815 PMC6786870

[B45] Tian X.-M. Wu Y. Yu Z. (2008). Isolation and screening of endophytes from *Artemisia annua* and their antagonistic activity to plant pathogens. *Acta Agric. Boreali-occidentalis Sin*. 17 186–190.

[B46] Vale P. F. McNally L. Doeschl-Wilson A. King K. C. Popat R. Domingo-Sananes M. R. (2016). Beyond killing: Can we find new ways to manage infection? *Evol. Med. Public. Health* 2016 148–157. 10.1093/emph/eow012 27016341 PMC4834974

[B47] Ventorino V. Parillo R. Testa A. Viscardi S. Espresso F. Pepe O. (2016). Chestnut green waste composting for sustainable forest management: Microbiota dynamics and impact on plant disease control. *J. Environ. Manag.* 166 168–177. 10.1016/j.jenvman.2015.10.018 26496847

[B48] Wagner M. R. Lundberg D. S. del Rio T. G. Tringe S. G. Dangl J. L. Mitchell-Olds T. (2016). Host genotypeand age shapethe leaf and root microbiomes of a wild perennial plant. *Nat. Commun*. 7:12151. 10.1038/ncomms12151 27402057 PMC4945892

[B49] Walters W. A. Jin Z. Youngblut N. Wallace J. G. Sutter J. Zhang W. (2018). Largescale replicated field study of maize rhizosphere identifies heritable microbes. *Proc. Natl. Acad. Sci. U.S.A.* 115 7368–7373. 10.1073/pnas.1800918115 29941552 PMC6048482

[B50] Wang Y. Yang Z. Luo S. Ding Q. Yang S. Wang K. (2022). Establishment of tissue culture regeneration system of *Artemisia Annua L*. *Mol. Plant Breed.* 13:43.

[B51] Wei Z. Yang T. Friman V. Xu Y. Shen Q. Jousset A. (2015). Trophic network architecture of root-associated bacterial communities determines pathogen invasion and plant health. *Nat. Commun.* 6:8413. 10.1038/ncomms9413 26400552 PMC4598729

[B52] Wu J. Ding W. Zhang Y. Guo W. (2007). Antifungal activities of primary extracts of *Artemisia annua* L. against two fungi. *Agrochemicals* 46 713–718. 10.3969/j.issn.1006-0413.2007.10.024

[B53] Xue C. Penton C. R. Shen Z. Zhang R. Huang Q. Li R. (2015). Manipulating the banana rhizosphere microbiome for biological control of Panama disease. *Sci. Rep.* 5:11124. 10.1038/srep11124 26242751 PMC4525139

[B54] Yang G. Li B. Zhang X. Wang Y. Li Q. Zhang W. (2009). Studies on flavonoids and their antioxidant Activities of *Artemisia annua*. *Chin. Herb. Med.* 33 1684–1688. 20218288

[B55] Yuan J. Zhao J. Wen T. Zhao M. L. Li R. Goossens P. (2018). Root exudates drive the soil-borne legacy of aboveground pathogen infection. *Microbiome* 6:156. 10.1186/s40168-018-0537-x 30208962 PMC6136170

