# Supplementary material for: An assembled bacterial community associated with Artemisia annua L. causes plant protection against a pathogenic fungus
Source: Front Microbiol. 2023 Oct 9;14:1218474. doi: 10.3389/fmicb.2023.1218474 (PMC10591200; doi:10.3389/fmicb.2023.1218474)
Supplement: Supplementary file 2 [file Data_Sheet_1.docx]

Supplementary materials


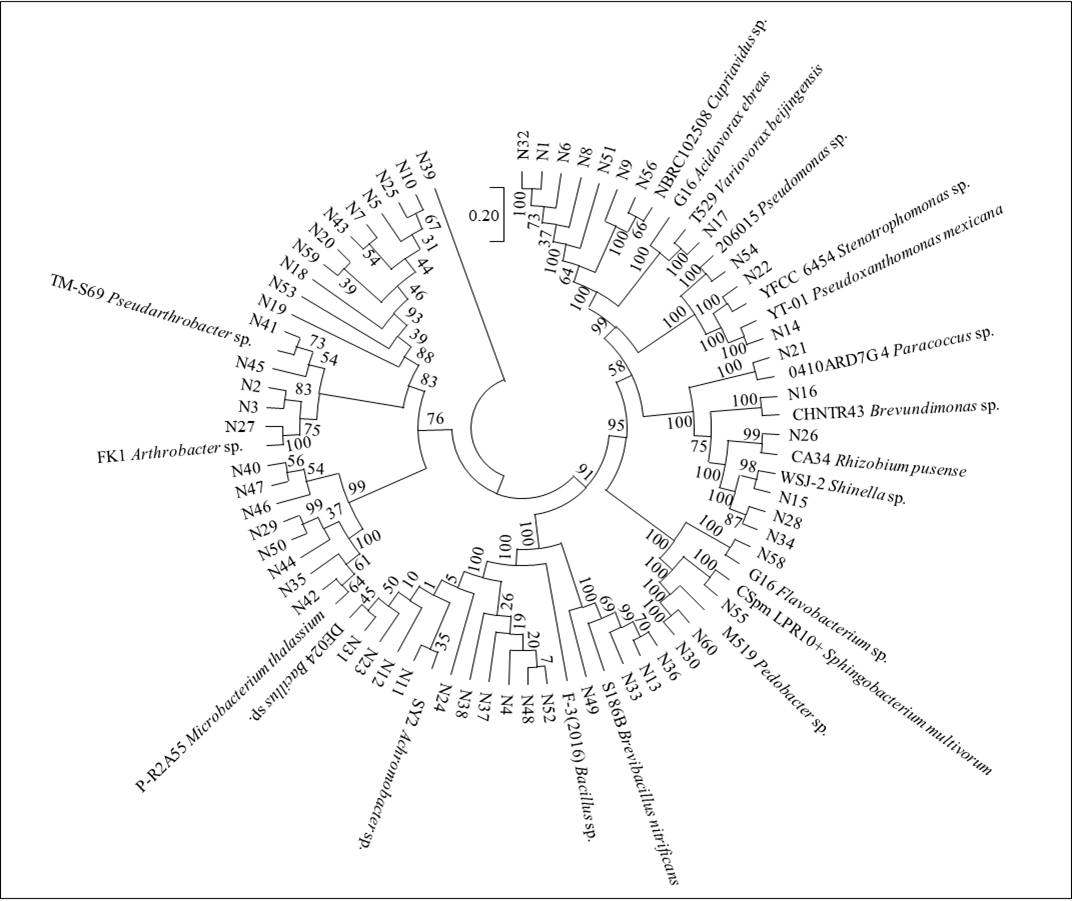


Supplementary Figure 1 Phylum of 60 endogenous bacteria strains


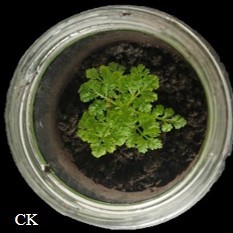

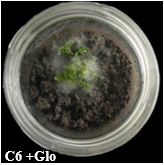

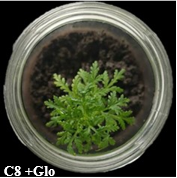


Supplementary Figure 2 Screening of bacteria synthetic communities against pathogenic fungus after inoculated with bacteria synthetic community and fungus *G. ultimum* var. for 7 days. CK was not treated with strains.

DNA extraction, amplification and sequencing of root endogenous bacteria

After the seedlings were cultured for seven days, under sterile condition, the container covers were opened, and then the seedlings were uprooted from soil respectively, the roots were washed cleanly using sterile water. The roots of three plants from each experiment were disinfected with 75% medical ethanol three times, 30 s once, and they were washed with 1x PBS solution 30 s, repeated three times, respectively. After the surface water was dry, the sterilized roots about 0.5 g were cut into small pieces, and ground with sterile quartz sand in liquid nitrogen, at last ground root samples were individually put into 2 ml sterile tubes and stored at -80℃ for bacterial DNA extraction.

The bacterial DNA of ground root samples was extracted with CTAB method. DNA integrity was examined through 1% agarose gel electrophoresis (AGE), the DNA concentrations of samples were quantified by Qubit 3.0 fluorescence quantitative instrument. In the first amplification, the barcoded primers F: CCTAYGGGRBGCASCAG and R: GGACTACNNGGGTATCTAAT were used to amplify V3-V4 region 16S rRNA of root bacteria. In the second amplification, Illumina Bridge PCR compatible primers were incorporated to amplify DNA sequence. Gene library size was detected by 2% AGE, the concentrations of gene library were determined using Qubit 3.0. The purified PCR products were sequenced on Illumina Novaseq6000 platform. All the extraction, amplification and sequencing procedures of root bacterial DNA were finished by Novogene Co., Ltd (Novogene Technology, Shanghai, China). The raw data were spliced and filtered to get clean data, and the clean data were denoised using DADA2 method, after the sequences with abundances less than 5 were filtered out, Amplicon sequence variants (ASVs, which were Corresponding to OTU) were got. The sequence alignment of bacterial 16S rDNA was conducted in the GenBank database of the NCBI website (http://rdp.cme.msu.edu/index.jsp).

Supplementary Table 2 ASV values of root endogenous eight-bacteria from *A. annua* seedling.

| Treatment |  | C8 | C8+Glo |
| --- | --- | --- | --- |
| DE024 *Bacillus* sp |  | 126.33 | 5.00 |
| S186B *Brevibacillus nitrificans* | | 794.33 | 879.00 |
| CHNTR43 *Brevundimonas* sp. | | 3.00 | 7.33 |
| CA34 *Rhizobium pusense* |  | 6105.33 | 7454.33 |
| 0410ARD7G4 *Paracoccus* sp. | | 191.00 | 8.00 |
| NBRC102508 *Cupriavidus* sp | | 6952.33 | 2189.67 |
| YFCC6454 *Stenotrophomonas* sp. | | 2196.67 | 56.67 |
| G16 Flavobacterium sp. |  | 1.00 | 1.67 |

Inhibitory effect of extract solution from the bacterial community C8 strains on *G. ultium*

Eight strains were cultured in NA liquid culture medium at 220 rpm at 37^o^C for 3 days. After the culture broth was centrifuged at 4000 rpm for 20 minutes, the supernatant was filtered, acidified with 1 mol/L HCl to pH 3.0, and then extracted with equal volume of each strain filtrate: methanol (1:1). After sufficient extraction, the 8 mixed solutions are concentrated at 50^o^C using a rotary vacuum evaporator. An equal amount of 8 strain extracts in methanol was dissolved and diluted to 2 mL, which was the reserve solution. Use. After filtered using membrane syringe with 0.22 μm aperture, and the sterilized stock solution was prepared. A hole was punched at the edge of the fungal colony using a 7 mm aperture punch, the fungal cake was moved to another PDA plate, and another hole 3 cm away from the fungal cake was made, which is the same size as the cake. 0.1 mL of bacterial extract was injected into the new hole, and negative and positive controls were set, three treatments, 3 repetitions each treatment, an equal amount of sterile water was added as the negative control, an equal amount of methanol as the positive control. the diameter of the antibacterial zone after culture 36 h was measured. It was shown that diameter of the antibacterial zone of the bacterial extract was significantly higher 37.5% than that of methanol, indicating that the bacterial extract had an inhibitory effect on the growth of the pathogenic *G. ultium*.

Supplementary Table 3 Inhibitory effection of extract solution from eight-bacteria C8 on fungal *G. ultium*.

| Treatments | Antibacterial circle diameter |
| --- | --- |
| Methanol | 0.73b±0.05 |
| Bacterial extract | 1.07a±0.05 |


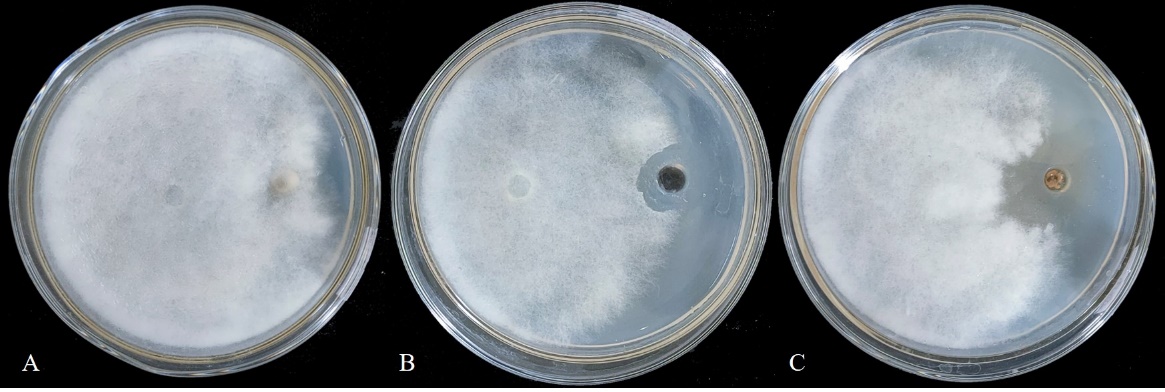


Supplementary Figure 3 Inhibitory assay of extract solution from eight-bacteria C8 on fungal *G. ultium*. Note: A represent the negative control with added water, B the positive control with added methanol, and C the experimental treatment with added bacterial extract.

Dual cultural assay a bacterial community C8-fungal interaction.

Dual culture assays of C8-fungus interactions were established in 9-cm diameter Petri dishes containing PDA medium. A 7-mm diameter plug cut from the edge of an actively growing colony of each test fungus was inoculated in the middle of each Petri dish. Fresh cells of the bacterial community C8 were streaked in 3-cm long parallel lines on either side of the fungal plug; the concentration of the bacterial community suspension was 10^6^ CFU/mL. Control plates with the fungus only were also conducted. After all treatments were incubated at 28^o^C in darkness for seven days, fungal hyphae challenged with the bacterial community C8 and from control plates were photographed. It is shown that there is significantly inhibitory effect of the bacterial community C8 on fungus *G. ultium.*


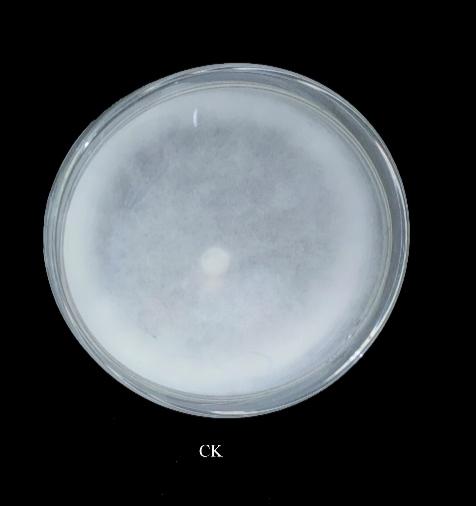

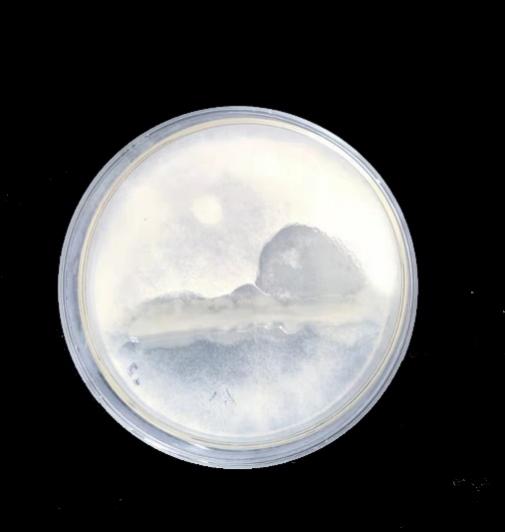


**B**

**A**

Supplementary Figure 4 Dual culture assay of a bacterial community C8-fungal interaction. A represents CK, B represents assembled eight-bacteria community.
